# Feasibility and short-term perioperative safety of composite deep cervical lymphatic reconstruction and vagus nerve microneurolysis in Parkinson’s disease: a first-in-indication pilot case series

**DOI:** 10.3389/fnagi.2026.1821749

**Published:** 2026-08-05

**Authors:** Haiwen Wang, Zetian Shi, Pengcheng Lv, Deqing Zeng, Haibo Wang, Xiongjun Mei, Shaokai Zhong

**Affiliations:** 1 Department of Microsurgery and Reconstructive Surgery, Dongguan Chashan Hospital, Dongguan, Guangdong, China; 2 Dongguan Key Laboratory of Microsurgery, Dongguan, Guangdong, China

**Keywords:** cervical lymphatic surgery, deep cervical lymphatic-venous anastomosis, meningeal lymphatics, Parkinson’s disease, perioperative safety, pilot study, surgical feasibility, vagus nerve

## Abstract

**Background:**

Parkinson’s disease (PD) remains associated with motor, non-motor, quality-of-life, and caregiver-burden needs despite pharmacological therapy. Anatomical and translational studies have drawn attention to cervical lymphatic pathways and vagal biology in PD, but surgical manipulation of these structures has not been clinically evaluated.

**Objective:**

To evaluate the technical feasibility and short-term perioperative safety of a composite cervical microsurgical procedure combining deep cervical lymphatic-venous reconstruction and cervical vagus nerve microneurolysis in selected patients with PD.

**Methods:**

In this prospective, single-center, single-arm, first-in-indication pilot case series, nine clinically diagnosed participants underwent the composite procedure. The main study focus was procedural completion and short-term safety monitoring. M3 was prespecified for exploratory medication-OFF scale collection. Outcome assessors were not blinded.

**Results:**

All nine participants completed the procedure and M3 assessment. Lymphatic reconstruction was completed in every case, with 10–12 anastomoses per participant, and intraoperative near-infrared indocyanine green imaging confirmed lymphatic-to-venous filling at completion. No surgery-related serious adverse events, reoperations, or complications requiring invasive intervention were recorded through available follow-up. Follow-up retention through M3 was 100%; lower denominators at later timepoints reflected staggered enrollment and follow-up maturity, not withdrawal or loss to follow-up. At M3, exploratory clinical scale changes were in the direction of score reduction. These descriptive observations are compatible with non-specific effects, including expectancy, natural fluctuation, medication influences, and observer bias, and cannot be attributed to the procedure without a control group, blinded assessment, standardized medication trajectories, or mechanistic readouts.

**Conclusion:**

The composite procedure was technically feasible, with no short-term surgery-related serious safety signal observed in this selected cohort. Exploratory clinical scale changes do not establish efficacy, durability, disease modification, target engagement, or component-specific effects. Controlled studies with blinded assessment and direct mechanistic endpoints are required.

## 1 Introduction

Parkinson’s disease (PD) is a progressive neurodegenerative disorder characterized by motor symptoms, non-motor symptoms, reduced quality of life, and increasing caregiver burden. Although dopaminergic therapies remain central to symptomatic treatment, many patients experience residual symptoms, motor fluctuations, and functional impairment despite pharmacological optimization.

Prior anatomical and preclinical studies have described meningeal lymphatic and glymphatic clearance pathways and their connections to cervical lymphatic structures (Louveau et al., 2015; Ding et al., 2021; Zou et al., 2019). In this pilot protocol, the deep cervical lymphatic region was selected as an operative region for feasibility evaluation. This background literature does not validate the intervention and does not establish that surgically altering cervical lymphatic outflow modifies PD symptoms, alpha-synuclein handling, or disease biology in humans. The present study was designed to test surgical feasibility and short-term perioperative safety, not to evaluate mechanistic hypotheses.

The cervical vagus nerve (VN) was included because it lies within the relevant cervical operative field and is biologically related to autonomic and gut-brain involvement in PD (Braak et al., 2003; Walter et al., 2018). In the present protocol, cervical VN microneurolysis was performed only as one component of a composite cervical microsurgical operation. This study was not designed to determine whether VN microneurolysis has an independent clinical effect or whether it is comparable or superior to non-invasive vagal stimulation approaches.

The aim of this first-in-indication pilot case series was to evaluate whether a composite cervical microsurgical procedure combining deep cervical lymphatic-venous anastomosis (DCLVA), lymph node-venous anastomosis (LNVA), and cervical VN microneurolysis could be completed in selected patients with PD and whether any short-term surgery-related serious safety signal was observed. Clinical rating scales were administered under standardized medication-OFF conditions as exploratory observations to inform the design of future controlled trials, not to establish clinical efficacy.

## 2 Materials and methods

### 2.1 Study design and setting

This was a prospective, single-center, single-arm, registry-based observational pilot case series evaluating the technical feasibility, perioperative safety, and exploratory clinical observations associated with a composite deep cervical lymphatic reconstruction procedure combined with cervical VN microneurolysis in patients with PD. The study was first-in-indication and feasibility-oriented; it was not designed or powered to test efficacy hypotheses. No control group, sham procedure, or blinded outcome adjudication was included. All clinical scale analyses were prespecified as descriptive and hypothesis-generating. Reporting follows the Strengthening the Reporting of Observational Studies in Epidemiology (STROBE) recommendations for observational studies (von Elm et al., 2007).

### 2.2 Ethics and informed consent

The study protocol was reviewed and approved by the Institutional Ethics Committee of Dongguan Chashan Hospital (Approval No. LLSC-XISXXM-2024015). All procedures were conducted in accordance with the principles of the 1964 Declaration of Helsinki and subsequent amendments. Written informed consent was obtained from all participants or their legally authorized representatives before enrollment and study-related procedures. Participant data were de-identified before analysis, and access to identifiable records was restricted to designated study personnel.

### 2.3 Participants

#### 2.3.1 Recruitment and diagnostic confirmation

Patients with a prior or suspected clinical diagnosis of PD who presented to Dongguan Chashan Hospital between March 2024 and April 2025 and expressed willingness to undergo deep cervical lymphatic reconstruction after clinical consultation were assessed for study eligibility. The cohort therefore represents a selected group of willing surgical candidates rather than a consecutively screened PD clinic population. Eligibility assessment was performed by a multidisciplinary team comprising neurology, microsurgery, and anesthesiology. Participants who met the clinical, surgical, and anesthetic eligibility criteria and provided written informed consent were enrolled.

PD diagnosis was confirmed by a neurologist with movement-disorder expertise according to the Movement Disorder Society (MDS) clinical diagnostic criteria for PD (Postuma et al., 2015), based on clinical phenotype and disease course. Dopamine transporter imaging and formal levodopa challenge testing were not protocol-mandated for all participants. Source records were re-reviewed after peer review to verify baseline phenotype, Hoehn-Yahr staging, and MDS-UPDRS Part III entries. One participant had an apparently discordant profile consisting of long disease duration, H-Y stage I, and a relatively high MDS-UPDRS Part III score; chart review indicated marked unilateral motor involvement as the likely explanation. Nevertheless, residual diagnostic uncertainty remains possible in a small clinically diagnosed cohort, and this limitation is explicitly acknowledged.

#### 2.3.2 Inclusion criteria

Participants were required to meet all of the following criteria:

(1) Confirmed diagnosis of PD according to MDS clinical diagnostic criteria, as assessed by a qualified neurologist with movement-disorder expertise;
(2) Cardinal motor features consistent with PD phenotype, including bradykinesia with resting tremor and/or rigidity, with or without postural and gait disturbance;
(3) Ongoing antiparkinsonian pharmacotherapy with clinically significant residual motor or non-motor symptoms and/or motor fluctuations despite at least one documented pharmacological optimization attempt;
(4) Ability to complete standardized medication-OFF assessments and scheduled rating scales;
(5) Willingness to undergo surgery and follow-up;
(6) Acceptable anesthesia risk
(7) Cervical anatomy and venous outflow conditions judged suitable for microsurgical reconstruction by the operating surgeon.

#### 2.3.3 Exclusion criteria

Participants were excluded for cognitive impairment likely to compromise reliable consent or scale assessment; prior deep brain stimulation or recent major neurosurgical intervention likely to confound the primary observation window; active cervical infection, cervical malignancy, or prior cervical surgery/radiation causing unsuitable anatomy; severe cardiopulmonary, renal, metabolic, or other systemic comorbidity precluding safe general anesthesia; uncorrectable coagulopathy or inability to safely manage anticoagulant or antiplatelet therapy; severe allergy to indocyanine green (ICG); anticipated inability to complete follow-up through M3; or severe psychiatric illness judged likely to compromise assessment reliability or perioperative safety.

### 2.4 Preoperative assessment

All participants underwent standardized preoperative evaluation comprising general medical assessment, anesthesia risk assessment, neurology assessment, and surgical feasibility assessment. General assessment included history-taking, medication reconciliation, physical examination, electrocardiography, complete blood count, serum biochemistry, coagulation profile, and thoracic imaging when clinically indicated. Neurology assessment included confirmation of PD phenotype, H-Y stage, disease duration, current pharmacological regimen, and planning of medication-OFF assessment procedures. Surgical feasibility assessment included evaluation of cervical anatomy, operative corridor, previous neck procedures or radiation, venous outflow conditions, positioning feasibility, and incision planning.

CSF or plasma alpha-synuclein assays, inflammatory biomarkers, and objective vagal function tests were not systematically obtained as protocol-mandated assessments. Therefore, this study cannot assess whether the procedure altered alpha-synuclein handling, inflammatory status, or vagal physiology.

### 2.5 Surgical intervention

#### 2.5.1 Operative setup, anesthesia, and patient positioning

All procedures were performed by microsurgeons experienced in lymphatic supermicrosurgery. Perioperative management was coordinated by the microsurgical, anesthesiology, and neurology teams, with attention to PD-related medication timing, airway precautions, and hemodynamic monitoring during cervical exposure and VN microneurolysis. Patients were positioned supine with mild neck extension and contralateral head rotation to optimize access to the posterior cervical operative field. Standard sterile preparation and draping were applied.

#### 2.5.2 Incision and surgical exposure

An initial 2–3 cm incision was made along the posterior border of the sternocleidomastoid muscle at the level of the Zone II–III junction (Robbins et al., 2008), individualized according to preoperative vascular marking and intraoperative fluorescence findings. The incision was extended when required to obtain safe exposure. This approach provided access to the cervical lymphatic collectors and adjacent superficial venous branches within the operative field, while allowing controlled dissection toward the carotid sheath when VN microneurolysis was performed. The subcutaneous tissue and platysma were divided in line with the incision, and the deep cervical fascia was opened carefully under magnification while protecting adjacent neurovascular structures.

#### 2.5.3 Lymphatic mapping

Low-dose indocyanine green (ICG) was administered intradermally or subcutaneously in cervical skin territories expected to drain toward the operative field. Near-infrared (NIR) fluorescence imaging was used to identify fluorescent lymphatic channels in real time and to guide the selection of candidate lymphatics for anastomosis. Intraoperative NIR-ICG findings were used only as a surgical mapping tool and as an immediate technical surrogate for lymphatic-to-venous filling after anastomosis. These findings should not be interpreted as evidence of long-term anastomotic patency, altered CNS lymphatic drainage, glymphatic transport, or disease-related target engagement.

#### 2.5.4 Microsurgical dissection and recipient vein selection

Under high-magnification operating microscopy, fluorescent or anatomically identifiable lymphatic channels within the operative field were dissected with care to avoid traction injury, thermal damage, and disruption of adjacent neurovascular structures. Encountered lymphatic outer diameters were approximately 0.1–0.8 mm. Recipient veins were selected in close proximity to the lymphatic channels. The external jugular vein or its tributaries were prioritized when suitable in caliber, wall quality, orientation, and proximity. When external jugular anatomy was limiting, alternative superficial venous branches within the same operative field were selected. Recipient vein selection and relevant intraoperative anatomy were documented in case report forms.

#### 2.5.5 Deep cervical lymphatic-venous anastomosis

Deep cervical lymphatic-venous anastomoses (DCLVAs) were performed between cervical lymphatic collectors and adjacent recipient venous branches using 12-0 nylon sutures with interrupted technique. End-to-end or end-to-side configuration was selected intraoperatively according to caliber matching, vessel orientation, and wall quality. Before anastomosis, recipient veins were gently irrigated with heparinized saline. A multi-bypass strategy was planned, with a target of at least 10 anastomoses per operative session when anatomically feasible. After each anastomosis, NIR-ICG re-imaging was used to assess immediate lymphatic-to-venous filling as an intraoperative technical patency surrogate. Additional sutures or technical adjustments were made when leakage, reflux, poor filling, or wall mismatch was observed.

#### 2.5.6 Lymph node-venous anastomosis

Lymph node-venous anastomosis (LNVA) was performed as an adjunctive component when an accessible cervical lymph node with suitable size, location, and tissue quality was identified within the operative field and a compatible recipient venous branch was available nearby. In this cohort, these anatomical criteria were met in all participants. The decision to proceed with LNVA was made intraoperatively by the operating surgeon based on local anatomy, lymph node accessibility, and recipient vein suitability. Because LNVA was performed as part of the composite procedural protocol, the present study cannot determine its independent contribution to any postoperative observation.

#### 2.5.7 Cervical vagus nerve microneurolysis

Cervical VN microneurolysis was performed during the same operative session when the carotid sheath could be safely exposed within the established surgical corridor. The VN was identified by its anatomical location between the common carotid artery and internal jugular vein. External microneurolysis was limited to careful release of perineural connective tissue or fascial bands that constrained safe exposure or mobilization. Dissection was performed under microscopic magnification using sharp technique, while avoiding intraneural dissection, direct nerve injury, excessive traction, electrocautery near the nerve, or thermal injury to adjacent structures.

The value of cervical VN microneurolysis as an independent intervention cannot be assessed in this study. No objective vagal function testing, such as heart-rate variability, baroreflex sensitivity, or high-resolution VN ultrasonography, was systematically performed before and after surgery. Therefore, no conclusion can be drawn regarding vagal target engagement, physiological modulation, or clinical contribution.

#### 2.5.8 Wound closure and postoperative monitoring

Following meticulous hemostasis, wounds were closed in layers. A closed-suction drain was placed routinely and removed within 24–48 h when output was minimal. A mildly compressive dressing was applied while avoiding excessive pressure over the anastomosis sites. Perioperative antibiotic prophylaxis followed institutional protocol. Postoperative monitoring focused on cervical hematoma, airway or swallowing compromise, wound complications, voice change, new neurological signs, hemodynamic instability, and need for unplanned intervention. Gentle cervical mobilization was initiated within 24–48 h postoperatively.

### 2.6 Follow-up schedule and follow-up maturity

Follow-up visits were prespecified at postoperative Month 1 (M1), M3, Month 6 (M6), Month 9 (M9), and Month 12 (M12). Prespecified assessment windows were M3, 90 ± 14 days; M6, 180 ± 21 days; M9, 270 ± 28 days; and M12, 360 ± 30 days. M1 was treated as an early observation visit with flexible scheduling. If multiple assessments occurred within a window, the evaluation closest to the target day was used for that timepoint.

Because participants underwent surgery on different dates in a rolling-enrollment pilot series, later follow-up windows had not matured uniformly by the fixed data cutoff. All nine participants reached and completed the M3 assessment window. Lower denominators at M6, M9, and M12 reflected that some participants had not yet reached the corresponding visit window by the data cutoff, rather than participant withdrawal, loss to follow-up, or adverse-event-related discontinuation. Later timepoint analyses therefore included only participants who had reached the relevant visit window and had paired baseline and follow-up observations. No imputation was performed.

### 2.7 Outcome measures

#### 2.7.1 Feasibility and safety outcomes

Procedural feasibility outcomes included completion of the planned composite procedure, completion of DCLVA and LNVA when anatomically feasible, number of lymphatic-venous anastomoses, intraoperative completion of cervical VN exposure and external microneurolysis when safely accessible, absence of unplanned conversion or procedure abortion, and immediate intraoperative NIR-ICG visualization of lymphatic-to-venous filling after anastomosis. NIR-ICG filling was treated as an immediate technical surrogate for intraoperative anastomotic function and was not interpreted as evidence of long-term patency or altered CNS lymphatic drainage.

Follow-up feasibility included completion of the prespecified M3 exploratory medication-OFF assessment. Later follow-up denominators were interpreted in relation to visit-window maturity at the fixed data cutoff.

Safety outcomes included adverse events and serious adverse events from surgery through the last available follow-up. Events of special interest included cervical hematoma, airway compromise, dysphagia, hoarseness or voice change, wound infection, skin-edge ischemia or delayed wound healing, venous thrombosis, lymphatic leakage, new sensory or motor neurological deficit, cardiovascular instability during or after VN exposure or microneurolysis, reoperation, and any complication requiring pharmacological, procedural, or invasive management. Surgical complications were categorized using the Clavien-Dindo classification when applicable (Dindo et al., 2004). Adverse events were also described using CTCAE v5.0 terminology when applicable.

#### 2.7.2 Standardized medication-OFF clinical assessments

Clinical rating scales were administered under standardized medication-OFF conditions. For participants receiving short-acting dopaminergic agents, medication was withheld for at least 12 h before assessment, consistent with established surgical trial methodology (Defer et al., 1999). For long-acting or extended-release formulations, withdrawal intervals were extended as clinically feasible and documented when possible. Adherence to withdrawal procedures was supported by nursing medication administration records and clinical review. When feasible, each participant was assessed by the same trained rater at a similar time of day to reduce intra-individual measurement variability.

Outcome assessments were performed by trained raters who were not blinded to participants’ surgical status. No centralized video-based blinded adjudication was performed. Consequently, all clinical scale observations are vulnerable to observer expectancy bias and are interpreted as exploratory only.

#### 2.7.3 Exploratory clinical scales

M3 was prespecified as the visit window for exploratory scale collection. MDS-UPDRS Part III in the medication-OFF state was the pre-designated exploratory motor scale (Goetz et al., 2008). Additional exploratory scales included MDS-UPDRS total score, Non-Motor Symptoms Scale (NMSS) total score (Chaudhuri et al., 2007), 39-item Parkinson’s Disease Questionnaire Summary Index (PDQ-39 SI) (Peto et al., 1995), and Caregiver Burden Inventory (CBI) total score (Novak and Guest, 1989). All scales share the same directional convention: higher scores indicate greater motor impairment, symptom burden, reduced quality of life, or caregiver burden. Changes were calculated as follow-up minus baseline; negative values indicate score reduction.

Published minimal clinically important difference (MCID) thresholds for MDS-UPDRS Part III and PDQ-39 SI were used only for descriptive individual-level contextualization, with exact Clopper-Pearson 95% confidence intervals (Clopper and Pearson, 1934; Horvath et al., 2015; Horvath et al., 2017). These thresholds were not used to adjudicate efficacy.

### 2.8 Concomitant antiparkinsonian medications

Participants continued their established antiparkinsonian pharmacological regimens during the study period. Dose modifications were discouraged unless clinically necessary; when identified during clinical follow-up, medication changes were recorded in the case report form by the treating neurologist. Structured levodopa equivalent daily dose (LEDD) trajectories were not systematically archived as a protocol-mandated endpoint, and complete quantitative LEDD time series are not available for the full cohort across all timepoints.

Clinical chart review did not identify documented addition or discontinuation of major antiparkinsonian medication classes during the primary M3 observation window. However, dose-level changes and complete LEDD trajectories were not prospectively captured, and medication-related confounding cannot be formally excluded.

### 2.9 Statistical analysis

All analyses were prespecified as descriptive and exploratory, consistent with the single-arm pilot design. No formal confirmatory efficacy testing was intended or performed. Continuous variables are summarized as mean ± standard deviation and median with interquartile range. Categorical variables are summarized as counts and percentages.

An available-case approach was applied at each timepoint: only participants with paired observations at baseline and the relevant follow-up were included in that timepoint’s analysis. Participants who had not yet reached a scheduled follow-up window by the data cutoff were considered not yet eligible for that timepoint, not lost to follow-up. No imputation was performed.

Two-sided paired Wilcoxon signed-rank tests were used to compare follow-up scores against baseline for each exploratory scale at each timepoint; when zero differences were present, the Pratt method was applied (Pratt, 1959). *P*-values are provided as descriptive references only; no multiplicity correction was applied. Hodges-Lehmann paired median differences with 95% bootstrap confidence intervals were used to summarize effect magnitude (Efron and Tibshirani, 1994). Because of the very small sample size and the discrete nature of exact signed-rank *p*-values, *p*-values should not be interpreted as confirmatory evidence. In particular, complete directional agreement across nine paired observations yields a small exact *p*-value by mathematical structure and does not establish treatment efficacy.

No *post-hoc* outlier-exclusion analysis was performed. Instead, all individual participant trajectories are displayed to allow transparent assessment of individual-level variability and the contribution of participants with high baseline burden.

### 2.10 Data management and quality control

Study data were recorded prospectively in standardized case report forms and entered into a dedicated database. Baseline characteristics, operative details, follow-up outcomes, and adverse events were cross-checked for completeness, internal consistency, and plausibility. Protocol deviations, including assessment-window deviations, were documented when applicable.

## 3 Results

### 3.1 Participant flow and retention

Nine eligible, willing surgical candidates with clinically diagnosed PD were enrolled after multidisciplinary assessment and written informed consent. All participants completed baseline medication-OFF assessment and underwent the planned composite procedure. All nine completed the prespecified M3 visit window assessment (9/9, 100%). No participant withdrew, declined further assessment, or was lost to follow-up before M3.

**FIGURE 1:**
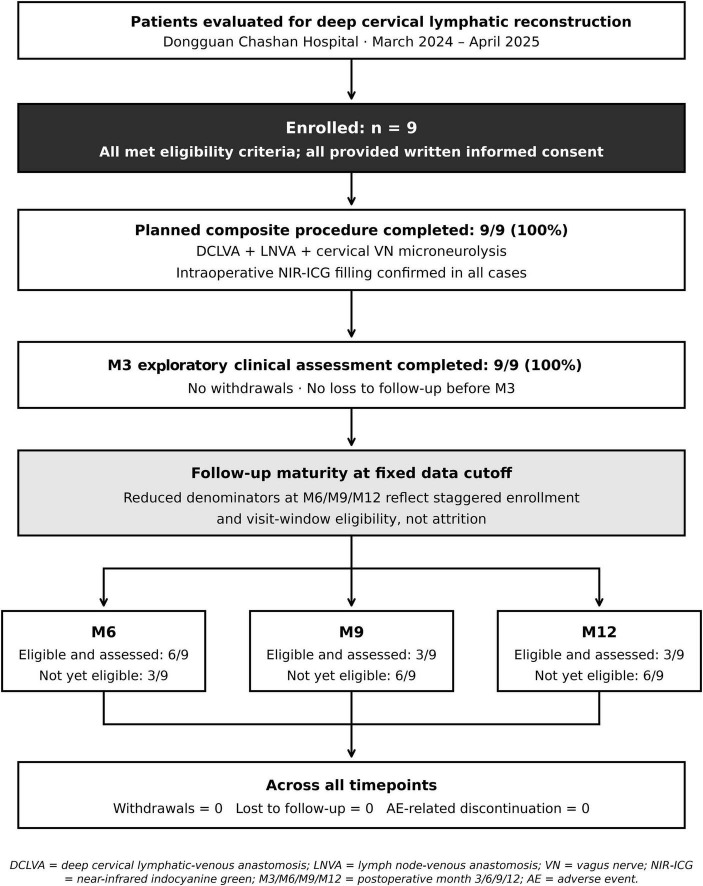
Participant flow and follow-up maturity at the fixed data cutoff. All nine participants completed the planned composite procedure and the prespecified M3 visit-window assessment. Reduced denominators at M6, M9, and M12 reflect staggered enrollment and visit-window maturity at the fixed data cutoff, not participant withdrawal, loss to follow-up, or adverse-event-related discontinuation.

The varying analytic denominators at M6, M9, and M12 reflect staggered enrollment and follow-up-window maturity at the fixed data cutoff, not participant attrition. Participants were enrolled between March 2024 and April 2025; those enrolled later had not yet reached the M6, M9, or M12 assessment windows by the data cutoff. Six participants had reached and completed the M6 window, and three had reached and completed both the M9 and M12 windows. No participant discontinued follow-up because of an adverse event. Later timepoints are therefore presented as descriptive available-case observations only, with no inference regarding durability. Participant flow and follow-up maturity are summarized in Figure 1.

### 3.2 Baseline characteristics

Baseline characteristics are summarized in Table 1. The cohort comprised six males and three females, with mean age 68.8 years (SD 7.2) and mean disease duration 8.7 years (SD 3.4). Hoehn-Yahr stage ranged from I to III. Baseline motor, non-motor, quality-of-life, and caregiver-burden scores were heterogeneous, consistent with the selected pilot nature of the cohort.

**TABLE 1:** Baseline characteristics (*n* = 9).

Case	Sex	Age (y)	Disease duration (y)	H-Y stage	MDS-UPDRS total	MDS-UPDRS part III (OFF)	PDQ-39 SI	NMSS total	CBI total
1	M	80	8	III	173	69	86	75	26
2	M	64	5	II	68	38	41	20	12
3	F	69	13	III	156	80	65	60	44
4	M	57	7	I	36	7	36	44	9
5	M	74	10	III	137	59	112	69	46
6	F	69	10	I	64	41	28	30	24
7	M	61	4	III	74	43	72	70	29
8	F	70	7	III	146	65	103	118	60
9	M	75	14	II	60	34	40	67	27
Mean ± SD	–	68.8 ± 7.2	8.7 ± 3.4	–	101.6 ± 50.8	48.4 ± 22.1	64.8 ± 30.7	61.4 ± 28.6	30.8 ± 16.5
Median [IQR]	–	69 [64–74]	8 [7–10]	–	74 [64–146]	43 [38–65]	65 [40–86]	67 [44–70]	27 [24–44]

All scales: higher scores = greater burden. H-Y, Hoehn-Yahr; MDS-UPDRS, Movement Disorder Society-Unified Parkinson’s Disease Rating Scale; PDQ-39 SI, 39-item Parkinson’s Disease Questionnaire Summary Index; NMSS, Non-Motor Symptoms Scale; CBI, Caregiver Burden Inventory; SD, standard deviation; IQR, interquartile range.

### 3.3 Procedural feasibility

All nine participants underwent the planned composite procedure without intraoperative conversion or procedure abortion. DCLVA was completed in all participants. LNVA was additionally performed in all participants because a suitable cervical lymph node and compatible recipient venous branch were identified within the operative field. The planned procedural target of at least 10 anastomoses was met in every participant, with 10–12 anastomoses performed per case. Intraoperative NIR-ICG fluorescence showed immediate lymphatic-to-venous filling after anastomosis completion in every case (Table 2).

**TABLE 2:** Procedural feasibility and short-term perioperative safety summary.

Domain	Measure	Result
Feasibility	Planned composite procedure completed	9/9
Feasibility	Intraoperative conversion or abortion	0/9
Feasibility	DCLVA and LNVA completed	9/9
Feasibility	Anastomoses per participant	10–12; protocol minimum ≥ 10 met in all cases
Feasibility	Intraoperative NIR-ICG lymphatic-to-venous filling confirmed	9/9
Retention	M3 assessment completed	9/9
Retention	M6 window reached by data cutoff	6/9
Retention	M9/M12 windows reached by data cutoff	3/9
Safety	Surgery-related serious adverse event	0/9
Safety	Reoperation or invasive intervention for complication	0/9
Safety	AE-related follow-up discontinuation	0/9
Safety	Mild cervical wound swelling (Clavien-Dindo Grade I)	4/9; resolved by POD 3–4
Safety	Retroauricular hypoesthesia, Grade I	2/9; no pain, motor deficit, or treatment required

DCLVA, deep cervical lymphatic-venous anastomosis; LNVA, lymph node-venous anastomosis; NIR-ICG, near-infrared indocyanine green; POD, postoperative day; AE, adverse event. Later follow-up denominators reflect eligibility at data cutoff, not loss to follow-up. Retroauricular hypoesthesia was anatomically consistent with possible greater auricular nerve stretch during cervical dissection.

These observations support the technical feasibility of performing the composite cervical microsurgical protocol in selected willing surgical candidates with PD. Intraoperative NIR-ICG filling was treated only as an immediate technical surrogate for anastomotic function; it does not establish long-term patency, altered CNS lymphatic drainage, glymphatic transport, or biological target engagement.

### 3.4 Safety outcomes

No surgery-related serious adverse events were recorded during the perioperative period or through available follow-up. No participant required reoperation or invasive intervention for a surgical complication. The prespecified events of special interest were actively monitored. No cervical hematoma requiring evacuation, wound infection requiring invasive management, venous thrombosis, lymphatic leakage, persistent dysphagia, persistent hoarseness, new focal neurological deficit, or cardiovascular instability requiring pharmacological or invasive treatment was observed.

Four participants developed mild cervical wound swelling, classified as Clavien-Dindo Grade I, which resolved within 3–4 days with conservative local care and without pharmacological, procedural, or invasive intervention. Two participants (Cases 6 and 8) reported retroauricular sensory hypoesthesia in the posterior auricular and lower pinna distribution, anatomically consistent with possible greater auricular nerve stretch during cervical dissection. No motor deficit, pain, or functional impact was recorded. Both sensory events were classified as Clavien-Dindo Grade I and CTCAE v5.0 Grade 1. No participant discontinued follow-up because of an adverse event.

A complete adverse-event listing with Clavien-Dindo and CTCAE v5.0 grading for all nine participants across all available follow-up visits is provided in Supplementary Table 1. These observations indicate that no short-term surgery-related serious safety signal was detected in this selected cohort. However, the sample size of nine is insufficient to detect uncommon, delayed, or cumulative complications, and prospective safety monitoring in larger studies remains essential.

### 3.5 Exploratory clinical scale observations at month 3

At M3, all nine participants had paired baseline and M3 observations. Within-participant score changes were in the direction of reduction from baseline across all five exploratory clinical scales. These findings are reported as descriptive observations only and cannot be attributed to the procedure in the absence of a control group or blinded assessment. They are also compatible with expectancy effects, natural fluctuation, regression to the mean, medication influences, observer bias, and intensified clinical contact.

Complete numerical summaries, including median changes, Hodges-Lehmann paired median differences, 95% bootstrap confidence intervals, and Wilcoxon *p*-values, are provided in Supplementary Table 2 for transparency and independent review. Group-level descriptive summaries and effect estimates are additionally presented in Supplementary Figures 1, 2. The *p*-value of 0.004 reported for the M3 scales reflects complete sign agreement across nine paired observations and carries no confirmatory inferential weight. Published MCID thresholds are listed in Supplementary Table 2 for individual-level descriptive context only and were not used to adjudicate efficacy.

**FIGURE 2:**
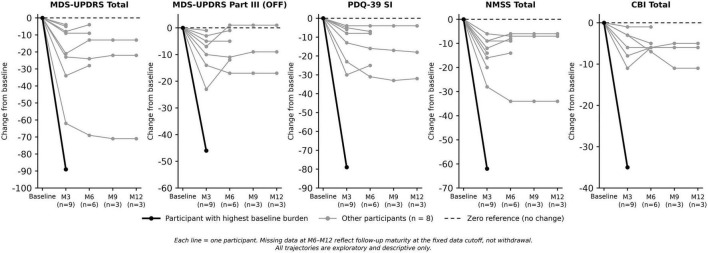
Individual participant trajectories for all five exploratory clinical scales from baseline through available follow-up. Each line represents one participant. Absent data points at M6–M12 indicate that the participant had not yet reached the corresponding visit window at the fixed data cutoff, not withdrawal or loss to follow-up. The horizontal reference line at zero indicates no change from baseline. All trajectories are exploratory and descriptive only and should not be interpreted as evidence of clinical efficacy, durability, or a procedure-related clinical effect.

Individual-level variability was substantial. One participant with the highest baseline burden showed larger absolute numerical changes than the remainder of the cohort. No outlier-exclusion analysis was conducted. Individual trajectories are displayed in Figure 2 to show all participants transparently without strengthening causal or efficacy inference.

### 3.6 Later exploratory observations

Available observations at M6 (*n* = 6), M9 (*n* = 3), and M12 (*n* = 3) are summarized in Supplementary Table 2 and displayed in Figure 2. These later observations are provided for descriptive longitudinal context only. They are not used to infer persistence or durability because not all participants had reached the corresponding follow-up windows by the fixed data cutoff, and the reduced denominators reflect follow-up-window maturity in a rolling-enrollment design, not loss to follow-up or adverse-event-related discontinuation. Therefore, later observations should not be interpreted as evidence of sustained improvement, durable benefit, or maintained recovery.

### 3.7 Representative clinical observations

Two cases are presented to illustrate individual-level heterogeneity, baseline-severity effects, and interpretive limitations in this unblinded pilot series. Case identifiers (A and B) do not correspond to Table 1 row order. These cases are descriptive clinical examples only and are not intended to establish treatment effect, durability, mechanism of action, or generalizability.

#### 3.7.1 Case A: high baseline burden and large numerical score changes

Case A was an older male participant (age 80 years; H-Y Stage III; disease duration 8 years; baseline MDS-UPDRS Total 173, MDS-UPDRS Part III 69, PDQ-39 SI 86) with high baseline clinical burden, severe medication-OFF motor disability, gait initiation difficulty, and substantial functional dependence. Lower clinical scale scores were recorded during available follow-up. This case represents the upper range of within-participant numerical change observed in the cohort and illustrates why individual trajectories are necessary in a very small case series.

**FIGURE 3:**
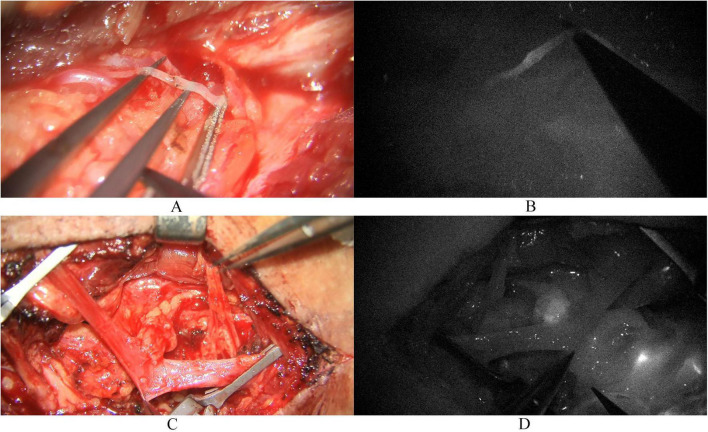
Intraoperative images of deep cervical lymphatic reconstruction in Case A. **(A)** Lymphatic-venous anastomosis completed. **(B)** ICG fluorescence imaging after lymphatic-venous anastomosis. **(C)** Lymph node-venous anastomosis completed. **(D)** ICG fluorescence imaging after lymph node-venous anastomosis.

Because the study lacked a control group and blinded assessment, the observed numerical changes cannot be separated from expectancy effects, natural fluctuation, regression to the mean, medication-related influences, perioperative factors, observer bias, or intensified clinical contact. Representative intraoperative images are provided in Figure 3, and a descriptive longitudinal gait video is provided as Supplementary Video 1. The video was not used for blinded endpoint adjudication and should be interpreted only as descriptive clinical documentation.

#### 3.7.2 Case B: early subjective sensory change and expectancy effects

Case B (male, 64 years; H-Y Stage II; disease duration 5 years; baseline MDS-UPDRS Total 68) reported subjective changes in olfactory and gustatory perception within 24 h after surgery, together with numerical score reductions at later visits. The early timing and subjective nature of this sensory report do not support a lymphatic-clearance mechanism and are compatible with expectancy or placebo effects in an unblinded surgical context.

**FIGURE 4:**
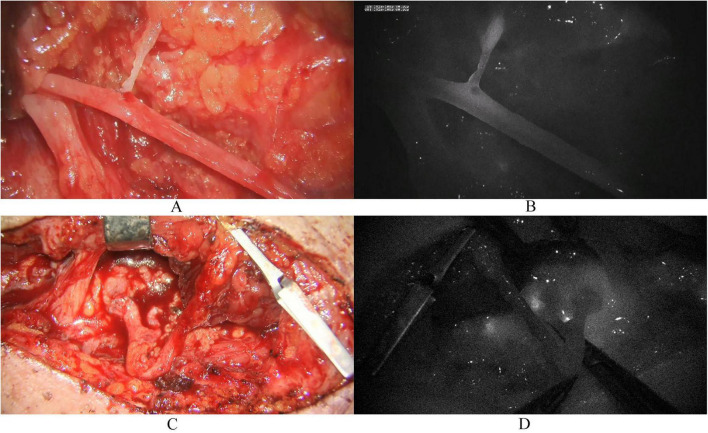
Intraoperative images of deep cervical lymphatic reconstruction in Case B. **(A)** Lymphatic-venous anastomosis completed. **(B)** ICG fluorescence imaging after lymphatic-venous anastomosis. **(C)** Lymph node-venous anastomosis completed. **(D)** ICG fluorescence imaging after lymph node-venous anastomosis.

Olfactory and gustatory changes were not assessed using validated objective olfactory or gustatory testing. Therefore, these observations should not be interpreted as evidence of treatment benefit, target engagement, or mechanism of action. This case is included to illustrate the interpretive risk of subjective outcomes in an uncontrolled surgical series. Representative intraoperative images are provided in Figure 4.

## 4 Discussion

### 4.1 Principal findings

This first-in-indication pilot case series shows that a composite cervical microsurgical procedure combining DCLVA, LNVA, and cervical VN exposure with external microneurolysis could be completed in nine selected willing surgical candidates with clinically diagnosed PD. The planned composite procedure was completed in all participants, the prespecified procedural target of at least 10 anastomoses was achieved in every case, and intraoperative NIR-ICG visualized immediate lymphatic-to-venous filling after anastomosis completion. No surgery-related serious adverse events, reoperations, or complications requiring invasive intervention were observed through available follow-up.

These feasibility and short-term safety observations constitute the main contribution of the study. Within-participant M3 clinical scale changes were observed in the direction of score reduction, but these findings are descriptive and hypothesis-generating only. The present design does not establish clinical efficacy, durability, disease modification, target engagement, or mechanism of action.

### 4.2 Interpretive boundaries of the exploratory clinical observations

The interpretation of clinical scale changes is substantially limited by the study design. This was a small, single-center, single-arm, unblinded case series without a sham or medical control group. Therefore, postoperative score changes cannot be distinguished from placebo effects, expectancy effects, regression to the mean, natural fluctuation of PD symptoms, observer bias, medication-related confounding, perioperative factors, postoperative care-related influences, or increased clinical attention (McRae et al., 2004).

The absence of blinded outcome adjudication is particularly important. Although assessments were conducted under protocol-specified medication-OFF conditions when feasible, MDS-UPDRS Part III remains susceptible to rater bias in an unblinded surgical context. Patient-reported and caregiver-reported outcomes are also vulnerable to expectancy effects. Future studies should incorporate blinded centralized video-based motor rating, blinded endpoint adjudication, objective motor and non-motor measurements, and prospective medication documentation at each visit.

### 4.3 Composite intervention and component attribution

The procedure evaluated here was deliberately composite. It included DCLVA, LNVA, cervical VN exposure, and external VN microneurolysis during the same operative session. The present design cannot determine whether any exploratory clinical observation, if reproducible under controlled conditions, relates to lymphatic-venous bypass, lymph node-venous anastomosis, cervical VN exposure, external microneurolysis, non-specific effects of peri-neural dissection, anesthesia, postoperative care, placebo response, or other factors.

Accordingly, the findings should be interpreted as observations after a composite procedural protocol rather than as evidence supporting any individual component. The study does not demonstrate that cervical VN microneurolysis is necessary, sufficient, or superior to non-invasive vagal stimulation approaches. It also does not demonstrate that lymphatic reconstruction altered CNS drainage. Future studies would require factorial, staged, or otherwise controlled designs to disentangle component-specific effects.

### 4.4 Biological rationale and untested mechanisms

The rationale for selecting the cervical lymphatic and vagal region was informed by anatomical, preclinical, and translational observations, including studies relating meningeal lymphatic drainage to PD and PD-like pathology (Ding et al., 2021; Zou et al., 2019). These studies motivated the anatomical region and composite protocol for feasibility evaluation only; they were not tested or confirmed by the present study.

No direct mechanistic readouts were obtained. Meningeal lymphatic flow imaging, DTI-ALPS indices, alpha-synuclein or neuroinflammation biomarkers, and objective vagal function measures were not systematically assessed. Intraoperative NIR-ICG visualized immediate anastomotic filling as a technical surrogate; it provides no information about CNS drainage, glymphatic transport, alpha-synuclein clearance, inflammatory modulation, or vagal physiology. Therefore, no inference about mechanism of action, target engagement, or biological pathway involvement should be drawn from the present uncontrolled human observations.

### 4.5 Follow-up maturity and later observations

All nine participants completed the M3 assessment. Later timepoints had smaller denominators because participants underwent surgery on different dates and not all had reached the M6, M9, or M12 visit windows by the fixed data cutoff. This structure reflects rolling enrollment and visit-window immaturity at the data cutoff, not participant withdrawal or loss to follow-up.

The incomplete maturity of later follow-up prevents inference regarding durability. Available later observations are useful only for descriptive longitudinal context and should not be interpreted as sustained improvement, maintained benefit, or durable clinical effect. Future studies should include a fixed enrollment period, predefined follow-up maturity requirements, and complete long-term follow-up before durability is assessed.

### 4.6 Medication-related confounding

Medication-related confounding could not be fully excluded. Participants continued established antiparkinsonian pharmacotherapy, and clinically necessary dose modifications were discouraged during the primary observation window. However, complete standardized levodopa equivalent daily dose (LEDD) trajectories were not prospectively archived for all participants, and formal LEDD-adjusted analyses could not be performed.

This limitation is material because PD motor and non-motor scores can be influenced by medication timing, dose adjustments, adherence, formulation changes, and withdrawal intervals before medication-OFF assessment. Future studies should prospectively record detailed antiparkinsonian medication regimens, calculate LEDD at each visit, standardize medication withdrawal intervals, and incorporate medication-adjusted sensitivity analyses.

### 4.7 Diagnostic certainty

Diagnosis was based on MDS clinical diagnostic criteria and specialist neurological evaluation. However, dopamine transporter imaging and formal levodopa challenge testing were not protocol-mandated for all participants. In a small surgical pilot cohort, diagnostic uncertainty in even a single participant may influence interpretation.

Source records were reviewed after peer-review critique, particularly for the participant with long disease duration, H-Y stage I, and a relatively high MDS-UPDRS Part III score. Marked unilateral motor involvement may explain this apparent discrepancy. Nevertheless, because DAT imaging or other supportive diagnostic testing was not universal, the cohort should be interpreted as a clinically diagnosed PD pilot cohort rather than a biomarker- or imaging-confirmed PD cohort. Future studies should incorporate stricter diagnostic confirmation, structured red-flag review, standardized neuroimaging review for mimics such as vascular parkinsonism and normal-pressure hydrocephalus, and dopamine transporter imaging where feasible.

### 4.8 Implications for future study design

The present study is most useful as a feasibility and short-term safety foundation for future controlled studies. It shows that the composite procedure could be completed in selected willing surgical candidates, that short-term surgical safety can be monitored using predefined adverse-event categories, and that exploratory clinical scales can be collected under medication-OFF conditions through M3.

The present case series provides three types of information for future study planning: (1) a procedural framework with predefined technical benchmarks, including a target of at least 10 anastomoses and intraoperative NIR-ICG visualization of lymphatic-to-venous filling; (2) descriptive variability estimates at M3 that may inform planning assumptions for future controlled trials; and (3) a medication-OFF, multi-scale assessment schedule that can be refined for future blinded and controlled studies.

Future studies should address the identified design gaps using a randomized or sham-controlled comparator where ethically and practically feasible, blinded centralized video-based MDS-UPDRS Part III rating, prospective medication documentation at every visit, and independent adverse-event adjudication. Direct mechanistic endpoint assessment, including cervical lymphatic patency imaging, glymphatic function indices, alpha-synuclein or neuroinflammatory biomarkers, and objective autonomic measures, would be required to determine whether the procedure engages any intended biological pathway. Component-specific designs, such as factorial, staged, or sequential arms, would be needed before any individual element of the composite procedure can be evaluated independently.

### 4.9 Limitations

This study has several important limitations. It was a very small, single-center, single-arm, unblinded pilot case series conducted in selected willing surgical candidates rather than in a consecutively screened general PD population. The absence of a sham or medical control group, blinded endpoint adjudication, standardized LEDD trajectories, and direct mechanistic readouts precludes inference regarding clinical efficacy, durability, disease modification, target engagement, or mechanism of action. The composite nature of the procedure also prevents attribution of any observation to DCLVA, LNVA, cervical VN exposure, external microneurolysis, or non-specific operative effects. In addition, later follow-up windows were not fully mature at the fixed data cutoff, diagnostic confirmation did not include universal dopamine transporter imaging or formal levodopa challenge testing, and several outcomes were subjective or semi-subjective. These limitations restrict generalizability and mean that the short-term safety observations cannot be extrapolated to uncommon, delayed, or larger-scale procedural risks.

## 5 Conclusion

In this selected nine-participant first-in-indication pilot cohort, composite deep cervical lymphatic reconstruction combined with cervical VN exposure and external microneurolysis was technically feasible, and no short-term surgery-related serious safety signal was observed through available follow-up. All clinical scale changes were exploratory and descriptive only. They do not establish clinical efficacy, durability, disease modification, target engagement, mechanism of action, or component-specific effects. These findings support only the rationale for future controlled studies with blinded assessment, prospective medication documentation, independent safety adjudication, and direct mechanistic endpoints.

## Data Availability

The original contributions presented in this study are included in the article/Supplementary material, further inquiries can be directed to the corresponding author.

## Supplementary material

Supplementary Figure 1 Descriptive mean ± SD changes from baseline across five exploratory clinical scales at M3, M6, M9, and M12 using available-case data. This figure is provided for orientation only; individual trajectories are displayed in main-text Figure 2. It should not be interpreted as evidence of efficacy or durability.

Supplementary Figure 2 Hodges-Lehmann paired median differences with 95% bootstrap confidence intervals at M3 (*n* = 9) and M6 (*n* = 6) for all five exploratory clinical scales. This figure is provided for statistical transparency only. The estimates are descriptive and should not be interpreted as confirmatory efficacy estimates in the absence of a control group, blinded assessment, or mechanistic readouts. HL, Hodges-Lehmann; CI, confidence interval.

Supplementary Video 1 Longitudinal gait observations under medication-OFF conditions in Case A, recorded at preoperative baseline, postoperative day 1, postoperative month 1, and postoperative month 11. The video is provided as descriptive clinical documentation only. It was not used for blinded endpoint adjudication and should not be interpreted as evidence of treatment efficacy, durability, or generalizability. Any apparent changes are subject to the same interpretive limitations as the other outcome observations in this uncontrolled pilot series.
